# Supplementary figures and images for: Land-use and socioeconomic time-series reveal legacy of redlining on present-day gentrification within a growing United States city
Source: PLoS One. 2025 Mar 3;20(3):e0317988. doi: 10.1371/journal.pone.0317988 (PMC11875366; doi:10.1371/journal.pone.0317988)

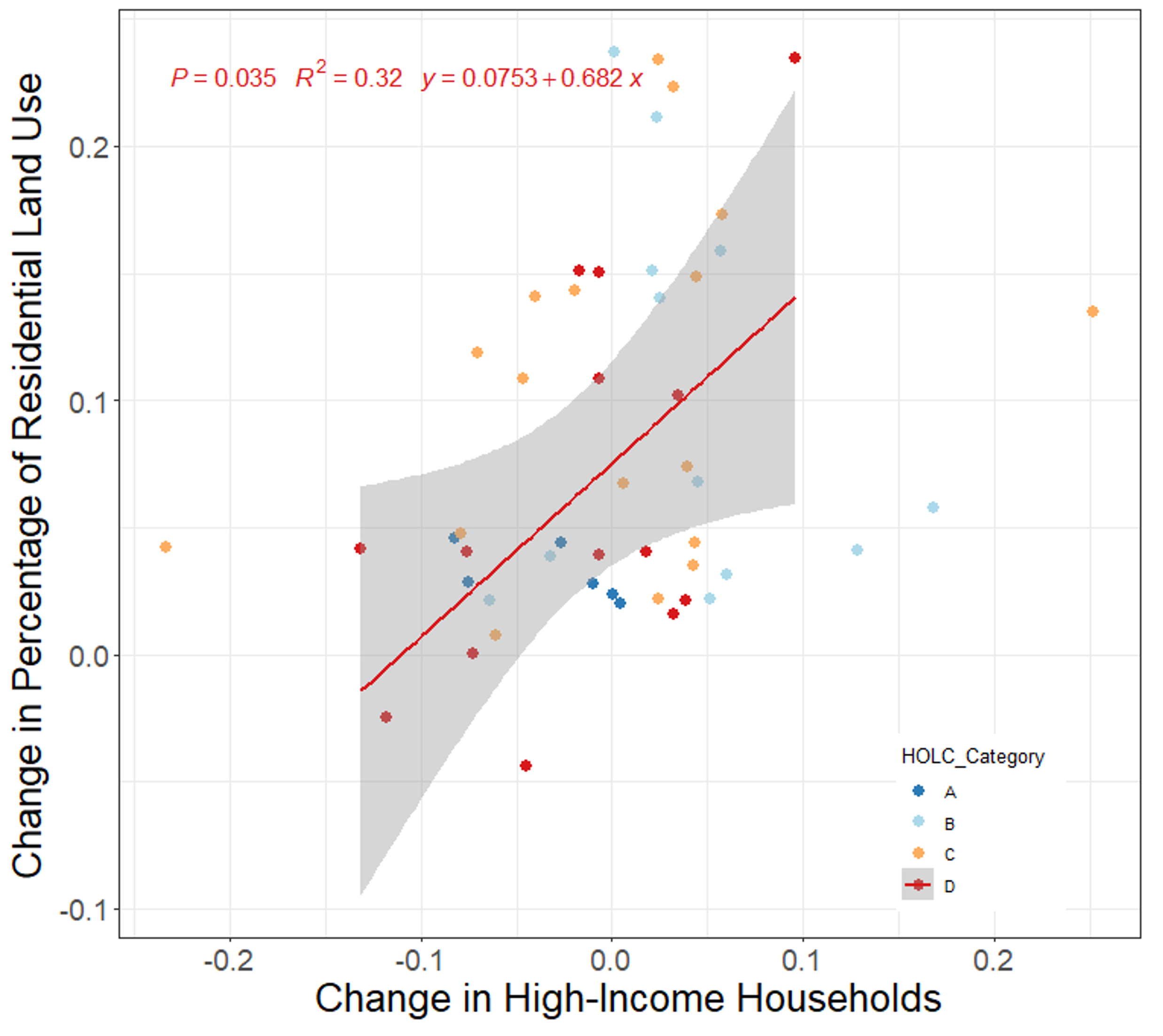

Supplement: S1 Fig — The 1997 and 2018 land-use data are from two different sources (1997: Drummond et al. 2019; 2018: Chen et al 2022) with differing classification methodologies. Only significant relationships have statistics and regression lines displayed. Shaded area represents the estimated standard error. (TIF) [file pone.0317988.s001.tif]

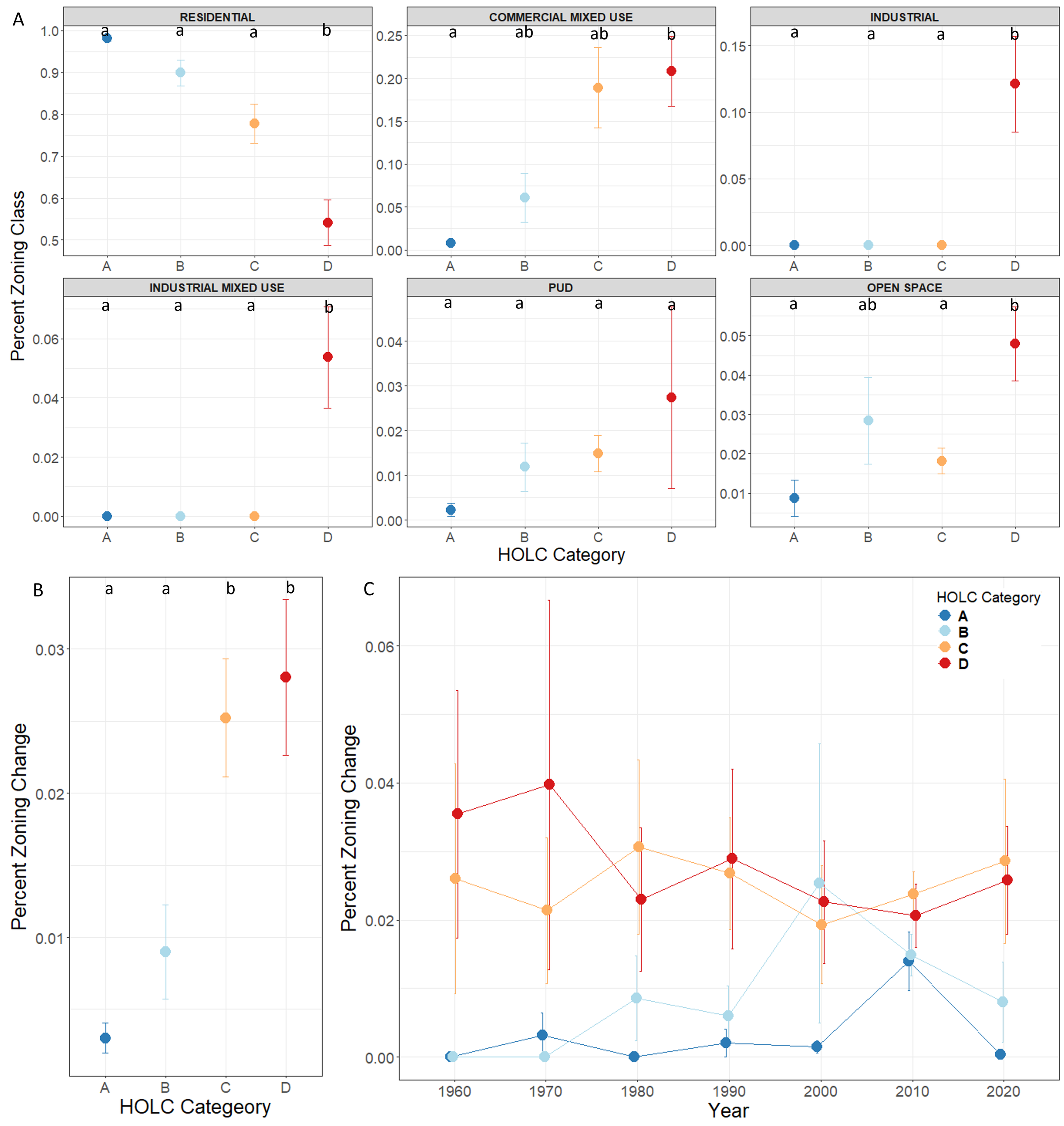

Supplement: S2 Fig — A: A comparison of the Zoning Class Percentages for six difference zoning designations across HOLC categories in the year 2020. B: The total area of parcels in the year 2020, which experienced a zoning change from 1955 to 2020. C: The total sum of area which experienced a zoning change across HOLC categories, aggregated to the decadal scale. The middle point corresponds with the data mean and whiskers corresponds to the standard error for data within the HOLC category and zoning variable. Significant differences between groups are represented by different letters above boxplots. (TIF) [file pone.0317988.s002.tif]
