# Supplementary material for: Land-use and socioeconomic time-series reveal legacy of redlining on present-day gentrification within a growing United States city
Source: PLoS One. 2025 Mar 3;20(3):e0317988. doi: 10.1371/journal.pone.0317988 (PMC11875366; doi:10.1371/journal.pone.0317988)
Supplement: Attachment — Submitted filename: PLOS_reviewer_comments_revised.docx [file pone.0317988.s003.docx]

We look forward to receiving your revised manuscript.

Kind regards,

Christopher A. Lepczyk
Academic Editor
PLOS ONE

Associate Editor:
General Comments
My apologies for the long delay in returning a decision on the manuscript. We went through an excessively large number of reviewers to obtain two qualified individuals to assess the work. As you will note, both reviewers found merit in the work and appreciated the level of analysis done. The main item from the reviews that warrants further consideration is the use/inclusion of zoning data. If zoning data are available for part or all of the time series, it would greatly aid your analyses and should be pursued. If these data are unavailable, then simply note that in a rebuttal. Finally, I would suggest including a paragraph in the Discussion that clearly describes all the limitations of the work.

Specific Comments
Abstract. Include main research questions/hypotheses.
L42. Change greenspace to green space here and throughout the ms.

Corrected
L46. One point that has come up that may want to note is that redlining was not the singular factor that has been recently described, but one of several that may co-occur, etc. You mostly note this point already, but may touch upon a bit more that it isn’t just a simple classification that explains the findings of other studies. Likewise, this point also relates to the reviewer’s comment about zoning.
 We have added a section focused on how zoning also plays a role in determining both the trend of homeownership and demographics, as well as the potential for HOLC C and D areas to be prime candidates for gentrification. However, as we explain in this section and in the associated figures, unfortunately the zoning data that is available is not usable to create a legitimate time-series. Additionally, we provide our reasons for using HOLC categories due to the fact they offer a standardized description of the various categories prior to subsequent lending impacts, and their fixed spatial extents provide a consistent location to analyze change over time. We do feel that by adding this section and highlighting that there is a greater flexibility in land use regulations in redlined districts, and that redlined districts are more vulnerable to shifts in zoning because they have fewer areas zoned single-family residential better make the case the HOLC boundaries were not a singular factor in driving land use and demographic trends, but are strongly linked to other regulatory processes.
L101-129. Much of this text can be moved to Methods or deleted. L101 should start the final paragraph that lays out the overarching goal/question followed by the specific questions/hypotheses/objectives.

We have incorporated this section into the Methods sub-section “Analysis of land-use diversity”, and removed any redundant text.
L130. I would suggest revising this first sentence a bit to indicate the gap in knowledge that you are seeking to answer.

The paragraph header has been adjusted to bring the main gaps in knowledge (the lack of time-series data, the importance of considering both land use and demographics) to the forefront before introducing our research questions.
L174. Please note here or elsewhere in the Methods what datum and projection were used in your geographic analyses.

Corrected
L192. How are you dealing with different resolutions of the datasets?

The resolution of the Drummond and Chen data sets are the same (1 m). We have edited the text to ensure that point is clear.
L215. Revise to ‘This approach…’

Corrected
L223. Did you evaluate collinearity amongst terms first? If not, please do. Also, did you have to transform any of the data? Finally, are you just using p-values here or AICc?

We have added text explaining how multicollinearity was expected between factors of race, education, and income. So we all demographic models were done independent from each other to isolate the specific demographic patterns.
L241. Here or elsewhere in Methods indicate the statistical software you used for analyses.

Corrected
L244. I would suggest cutting this sentence. Results here should be very succinct. You found x relationship, with y parameters being important.

Corrected
Fig. 2 legend. Delete ‘We determined significance through a generalized linear model with interactions between HOLC category and years.’ Also, no description of panel B is noted.

Corrected.
L276. Can revise to just indicate result, do not need to describe the statistical approach used as that was presented in the Methods.

We have removed superfluous text which was already described in the Methods section
L277. Provide the full statistical results (F = xxx, DF = x,y, p = z.zz) here and elsewhere in Results. Also, delete the sentence that starts at this line as you already explained post hoc approach in Methods.

Corrected and appreciated! On producing the statistics the authors discovered a typo in the statistical code, when corrected we found that HOLC D was significantly different in Shannon Diversity back to 1937. This actually bolsters our argument that redlining practices were not the creative force for present day land use segregation, but did correlate with increases in segregation of land use diversity in the following decades. We have corrected this text here and in the discussion.
L290. See previous comment on including detailed stats.
L301, L315, L358. Provide the statistical details of the analyses here.

We have added statistical details for all linearly modeled relationships to the text
Fig. 3 legend. Delete ‘We determined significance through a generalized linear model with interactions between HOLC category and years.’

Corrected
Fig. 4 legend. Delete ‘We determined significance through a generalized linear model with interactions between HOLC category, income class, and years.’

Corrected
L354. Move this item to Methods.

We have moved to the methods and added text on how we conducted the analysis.
Fig. 5 legend. Delete ‘We determined significance through a generalized linear model with interactions between HOLC category and years.’ And ‘Regression line equations and p values by HOLC category are provided.’

Corrected
L374. Can cut most of first two sentences. Begin with answers to your questions.
 Corrected

[Note: HTML markup is below. Please do not edit.]

Reviewers' comments:

Reviewer's Responses to Questions

**Comments to the Author**

1. Is the manuscript technically sound, and do the data support the conclusions?

The manuscript must describe a technically sound piece of scientific research with data that supports the conclusions. Experiments must have been conducted rigorously, with appropriate controls, replication, and sample sizes. The conclusions must be drawn appropriately based on the data presented.

Reviewer #1: Partly

Reviewer #2: Yes

2. Has the statistical analysis been performed appropriately and rigorously?

Reviewer #1: Yes

Reviewer #2: Yes

3. Have the authors made all data underlying the findings in their manuscript fully available?

The [PLOS Data policy](https://gcc02.safelinks.protection.outlook.com/?url=https%3A%2F%2Furldefense.com%2Fv3%2F__http%3A%2F%2Fc05y1x9s.r.us-east-2.awstrack.me%2FL0%2Fhttp%3A*2F*2Fwww.plosone.org*2Fstatic*2Fpolicies.action*23sharing%2F1%2F010f0191ba754921-30047aee-024e-480c-a811-c18971e04b1c-000000%2FBVeokFYlxU_-zMcmb9HRNKJemHY%3D175__%3BJSUlJSU!!Mak6IKo!OcFtLTvq55FlZ07cL4KFpsS8EJgEgDwCFZWTVPmz6OYupz88TWATQJqT_5CEseXq3xEA0aQsp2tIdlQpfQE%24&data=05%7C02%7Cpibsen%40usgs.gov%7Ca2a4c556dc1f4d3eabfd08dcd251e074%7C0693b5ba4b184d7b9341f32f400a5494%7C0%7C0%7C638616496374290439%7CUnknown%7CTWFpbGZsb3d8eyJWIjoiMC4wLjAwMDAiLCJQIjoiV2luMzIiLCJBTiI6Ik1haWwiLCJXVCI6Mn0%3D%7C0%7C%7C%7C&sdata=5eJFVr2t22eecvbJn7x2hyry5ZEkSdRatSzU9XbUkQ8%3D&reserved=0) requires authors to make all data underlying the findings described in their manuscript fully available without restriction, with rare exception (please refer to the Data Availability Statement in the manuscript PDF file). The data should be provided as part of the manuscript or its supporting information, or deposited to a public repository. For example, in addition to summary statistics, the data points behind means, medians and variance measures should be available. If there are restrictions on publicly sharing data—e.g. participant privacy or use of data from a third party—those must be specified.

Reviewer #1: Yes

Reviewer #2: Yes

4. Is the manuscript presented in an intelligible fashion and written in standard English?

PLOS ONE does not copyedit accepted manuscripts, so the language in submitted articles must be clear, correct, and unambiguous. Any typographical or grammatical errors should be corrected at revision, so please note any specific errors here.

Reviewer #1: Yes

Reviewer #2: Yes

5. Review Comments to the Author

Please use the space provided to explain your answers to the questions above. You may also include additional comments for the author, including concerns about dual publication, research ethics, or publication ethics. (Please upload your review as an attachment if it exceeds 20,000 characters)

Reviewer #1: The principal claim of this article is succinctly summarized in the title: “Land use and socioeconomic time-series reveal legacy of redlining on present-day gentrification within a growing United States city,” but the study asks and answers much more far-reaching claims than this. And, the study effectively qualifies the specific role of “redlining,” meaning the impact of the 1938 HOLC Security Maps as a causal factor in this overall process. Instead, the authors use the HOLC categories as a snapshot of the land use and demographic conditions at the starting-point in their longitudinal study. The goal at the heart of the study is to track long-term changes in land use diversity and demographic areal composition from 1937 to 2019. While the long-term correlations between HOLC categories (developed originally and used by lending agencies thereafter for the purpose of qualifying applicants for mortgages) and downstream social inequality, urban amenities, environmental injustice, and other factors has been well-documented now in numerous studies amply cited in this study, the key innovation here is the use of an allegedly unique—for Denver-- high-resolution land-use data series (Drummond et al 2019) , for Denver in the years 1937, 1957, 1977, and 1997. This data series allows the authors to a) set a benchmark of land use prior to any possible impact of the HOLC maps, and b) track changes in 20-year intervals between the onset of the redlining era and pour contemporary, particularly the first two decades of the 21st century.
In the authors’ own words, “The spatial patterns of this connection between the built environment, past discriminatory policies, and the potential vulnerability to neighborhood gentrification have been connected from the present to the HOLC boundaries (30), but to understand how these spatial patterns emerged over time, time series data is needed. (96-101). The authors address three specific questions: “1. How have patterns in land use and demographics developed and diverged in formally categorized HOLC areas across Denver from 1937 to 2019? 2. How has the diversity of demography in Denver neighborhoods connected to HOLC areas changed over the same period? 3. How have patterns of inequality in land use and demographics from the previous decades influenced rapid present-day changes in Denver’s demographics and resulting neighborhood-specific patterns in gentrification?” (129-138).

Overall the authors are successful in answering all three of these questions, but there is a major factor that they both recognized and fail to include in their analyses, which calls into question the whole rationale for depending on HOLC classifications in the first place: Denver’s zoning ordinances.

The authors do show statistically significant patterns in which the land-use diversity was reinforced in HOLC category D areas across the entire time period, which residential, single-family land use also increased in areas A and B. Proportions of college-educated, white, and higher-income households were all significantly lower for HOLD D areas than for HOLC-A areas over time. Given the goals of the study, a significant achievement is that the authors are able to show significant changes within the overall trends from 1937-2019 in two time-periods: 1) a significant impact of urban renewal policies and projects during the 1960-1980 interval, and 2) the impact of gentrification in areas previously disadvantaged by the longer-term trends, in the early 20th century period.

While all of this is very solid statistically, this reviewer believes that the authors made a critical methodological mistake by not identifying and including the geographic boundaries of the city’s zoning ordinances, along with the land-use restrictions codified by those ordinances for those geographic boundaries, as independent variables in addition of the HOLC boundaries. The HOLC boundaries, after all, were established for the single purpose of evaluating the loan-security risk of residential properties. Thereafter, they were used or adapted by banks for decades, but only for the purpose of home loan qualifications. The principal innovation of this study is to use the Drummond, et al land-use maps, which capture all categories of land use. The authors use these maps primarily along the dimension of “diversity,” which is essentially bi-variate in their usage: they seek to distinguish high-diversity areas (where industrial and other non-residential uses are concentrated), and low-diversity, where primarily residential areas are concentrated. Zoning ordinances were invented in the early 20th century to exclude industrial and other commercial uses from certain residential areas. (and also to optimize industrial development). They did not exclude residential development within industrial/commercial areas, but they did exclude industrial/commercial development in areas newly developed to be exclusively residential. This all happened concurrent with the rise of racial restrictions, usually to exclude non-whites from those same all-residentially zoned areas. While this reviewer is not familiar with Denver’s specific zoning ordinances, based on their use in cities with which the reviewer does have familiarity, it is nearly certain that they were used to exclude land use “diversity” from residential areas over this long period. It is also likely that they were revised significantly over the years. It is also certain that zoning ordinances have a direct impact on land use, while HOLC categories have only an indirect impact. And, it is very well known that residential property values are directly impacted by proximity to nuisances typical of industrial and commercial land uses. It is likely, therefore, that the exclusivity of HOLC areas A and B, and the land use diversity of areas C and D, can be explained entirely by the regulatory effect of these ordinances. The word “zoning” only appears three times in this manuscript (95, 403, 416), but in each of these passages the authors acknowledge the importance of zoning ordinances.

The authors laudably avoid assigning HOLC maps a singular causal role in the longer temporal patterns of land use and demographic spatial inequality. They show empirically that the inequalities of land use and demographic geography pre-existed the HOLC maps, and that changes occurred long after their implementation. This reviewer believes that the failure to consider the role of zoning geographies across this period is a major oversight. While the HOLC maps have come to stand for a long-term inscription of inequality in urban landscapes, the authors needn’t have tied their study so closely to those maps which, as they acknowledge, do not even cover downtown areas. The status quo of land use and social demography in 1937 and subsequent years could have been classified by an independent set of area units, and tracked over time for long-term patterns. The use of zoning geographies would have gone a long way toward helping to explain those patterns, as city planning authorities were required to certify all building project according to these maps—and not to the HOLC maps. This reviewer does not recommend removing the HOLC geographies from the study—far from it: the inclusion of the HOLC geographies and categories should still be an important set of variables in the study. But they should be used in conjunction with the explanatory power of the zoning geographies. This approach will a) give a much more accurate view of the long-term urban developmental historical process (recognizing the factor most likely to shape land use), and b) help to advance the latest state of the art in understanding the impact of the HOLC maps. My recommendation, therefore, does not imply scrapping the entire approach of this study. Instead, it suggests the addition of mapping layers with associated attributes, most probably chosen to coincide with the four land-use data points of 1937, 57, 77, and 97, and re-running their analyses to include the independent variable of land use restrictions on every space in the study.

We greatly thank Reviewer 1 for their in-depth consideration of this work. We also completely agree with them! Previously we had not included any analysis on zoning (aside from the comments we had already made expressing that zoning ordination would also be considered a driving force behind patterns in land use configuration and diversity. This was borne out of a limited access to historical zoning data. However, thanks to this comment we have engaged with the GIS office of the City of Denver and received some historical zoning data. However, this data only goes back to 1955, and only indicated the year when a zoning ordinance was changed, but it does not indicate exactly what the previous ordinance was. Thus, we cannot exactly track a similar time-series of zoning influence on land use as we do with the rest of the analysis.

However, we have added a section focused on how zoning also plays a role in determining both the trend of homeownership and demographics, as well as the potential for HOLC C and D areas to be prime candidates for gentrification. However, as we explain in this section and in the associated figures, unfortunately the zoning data that is available is not usable to create a legitimate time-series. Additionally, we provide our reasons for using HOLC categories due to the fact they offer a standardized description of the various categories prior to subsequent lending impacts, and their fixed spatial extents provide a consistent location to analyze change over time.

With the limited zoning data we have we were able to present some analysis that reflects similar patterns to what we have shown in regards to land cover change. Firstly, we have found that former HOLC D areas currently are zoned to include a greater diversity of zoning ordinances, and moreover, by tracking the dates of zoning change, we found HOLC C and D areas experienced more regular changes in their zoning ordinances over a period of 1955-2020. These results are now included as Supplemental Figure 2.

We do feel that by adding this addition text, analysis, and highlighting that there is a greater flexibility in land use regulations in redlined districts, and that redlined districts are more vulnerable to shifts in zoning because they have fewer areas zoned single-family residential better make the case the HOLC boundaries were not a singular factor in driving land use and demographic trends, but are strongly linked to other regulatory processes.

Reviewer #2: This research analyzed changes in land use and socioeconomic factors, considering the impacts of historical redlining, to identify current patterns of gentrification in Denver, USA. They used boxplots and statistical analysis to test three research questions developed in this research. Given the increasing academic interest in the lingering impacts of redlining on urban dynamics, the findings here are noteworthy and provide some implications. Before publication, the manuscript needs to address the following issues.

Major issue
Introduction
1. Although the authors noted some critiques of using HOLC data in the first paragraph (e.g., over-causality), it would be beneficial to also address another criticism, such as the skepticism regarding the actual impact of HOLC (check this:
[https://doi.org/10.1177/0096144203029004002](https://gcc02.safelinks.protection.outlook.com/?url=https%3A%2F%2Furldefense.com%2Fv3%2F__https%3A%2F%2Fdoi.org%2F10.1177%2F0096144203029004002__%3B!!Mak6IKo!OcFtLTvq55FlZ07cL4KFpsS8EJgEgDwCFZWTVPmz6OYupz88TWATQJqT_5CEseXq3xEA0aQsp2tICNEmJd0%24&data=05%7C02%7Cpibsen%40usgs.gov%7Ca2a4c556dc1f4d3eabfd08dcd251e074%7C0693b5ba4b184d7b9341f32f400a5494%7C0%7C0%7C638616496374297534%7CUnknown%7CTWFpbGZsb3d8eyJWIjoiMC4wLjAwMDAiLCJQIjoiV2luMzIiLCJBTiI6Ik1haWwiLCJXVCI6Mn0%3D%7C0%7C%7C%7C&sdata=ga7qwZTJvWvblixuGCjd3RZn4q4CqMhimG5%2BFKco9ps%3D&reserved=0)).

Thank you, we have cited Hilliers work in the introduction, and we have added text in the intro describing her central thesis that HOLC mapping did not cause segregation at the neighborhood scale, but may have reinforced the practice at the individual household level. We have also added text in the conclusion describing how our results have added evidence to Hillier’s theory, as we found land-use and demographic segregation both pre-existing HOLC maps, and growing in the following decades.

2. More recently published papers should be cited in lines 88-90.
[https://doi.org/10.1016/j.landurbplan.2024.105019](https://gcc02.safelinks.protection.outlook.com/?url=https%3A%2F%2Furldefense.com%2Fv3%2F__https%3A%2F%2Fdoi.org%2F10.1016%2Fj.landurbplan.2024.105019__%3B!!Mak6IKo!OcFtLTvq55FlZ07cL4KFpsS8EJgEgDwCFZWTVPmz6OYupz88TWATQJqT_5CEseXq3xEA0aQsp2tI3IkNKLA%24&data=05%7C02%7Cpibsen%40usgs.gov%7Ca2a4c556dc1f4d3eabfd08dcd251e074%7C0693b5ba4b184d7b9341f32f400a5494%7C0%7C0%7C638616496374303837%7CUnknown%7CTWFpbGZsb3d8eyJWIjoiMC4wLjAwMDAiLCJQIjoiV2luMzIiLCJBTiI6Ik1haWwiLCJXVCI6Mn0%3D%7C0%7C%7C%7C&sdata=lS89H6aqgYV4JTamIlUge4gkLVIiKYpe07ZFxFfT7Cs%3D&reserved=0)
[https://doi.org/10.1016/j.landurbplan.2024.105028](https://gcc02.safelinks.protection.outlook.com/?url=https%3A%2F%2Furldefense.com%2Fv3%2F__https%3A%2F%2Fdoi.org%2F10.1016%2Fj.landurbplan.2024.105028__%3B!!Mak6IKo!OcFtLTvq55FlZ07cL4KFpsS8EJgEgDwCFZWTVPmz6OYupz88TWATQJqT_5CEseXq3xEA0aQsp2tI6RP3cLI%24&data=05%7C02%7Cpibsen%40usgs.gov%7Ca2a4c556dc1f4d3eabfd08dcd251e074%7C0693b5ba4b184d7b9341f32f400a5494%7C0%7C0%7C638616496374309877%7CUnknown%7CTWFpbGZsb3d8eyJWIjoiMC4wLjAwMDAiLCJQIjoiV2luMzIiLCJBTiI6Ik1haWwiLCJXVCI6Mn0%3D%7C0%7C%7C%7C&sdata=f2u5a1ClrrUnZmiugbv21vqdigdPB1DB8DMU3h%2FX1bI%3D&reserved=0)
[https://doi.org/10.1080/13549839.2024.2380854](https://gcc02.safelinks.protection.outlook.com/?url=https%3A%2F%2Furldefense.com%2Fv3%2F__https%3A%2F%2Fdoi.org%2F10.1080%2F13549839.2024.2380854__%3B!!Mak6IKo!OcFtLTvq55FlZ07cL4KFpsS8EJgEgDwCFZWTVPmz6OYupz88TWATQJqT_5CEseXq3xEA0aQsp2tI84AjZA4%24&data=05%7C02%7Cpibsen%40usgs.gov%7Ca2a4c556dc1f4d3eabfd08dcd251e074%7C0693b5ba4b184d7b9341f32f400a5494%7C0%7C0%7C638616496374316610%7CUnknown%7CTWFpbGZsb3d8eyJWIjoiMC4wLjAwMDAiLCJQIjoiV2luMzIiLCJBTiI6Ik1haWwiLCJXVCI6Mn0%3D%7C0%7C%7C%7C&sdata=hI8xZJEh%2BDnUK0FvBCLCTOVP3h5K1DVb4DOykNTJjFE%3D&reserved=0)

Thank you for the recent references. These are all great additions, and I am quite fond of the Jung 2024 paper as well.

3. The first and second research questions should be revised for clearer distinction. Currently, they have some overlap.

The reviewer makes a good point. We have condensed our research questions to two key questions, one focused on land use and demographic patterns through time, and one focused on the resulting gentrification pattern.

Methods
1. The authors used the Shannon-Wiener index (H') to calculate the compositional features of land use. Are there other metrics or methods that can assess the configurational aspect of land use? Even if two districts have similar compositional attributes, their configurational characteristics may differ significantly, and these can provide some information.

Thank you for the comment. While there are other land use diversity metrics that account for spatial configuration, such as the Land Use Diversity Index, we purposely selected Shannon for its resistance to configuration change. For this study we are concerned with the overall compositional land cover diversity and change over time. While including configurational change in this analysis would provide information about how land use is arranged in neighborhoods, the literature does not draw many relationships between redlining practices and land use configuration. Therefore, including configuration as a metric could result in false positives in differences in neighborhoods that might not be related to other urban planning practices. We have added text in the introduction further explaining our rationale for using H’ as our diversity index.

2. Why do the authors use a quasi-binomial distribution to fit their models? Providing a rationale for this choice would strengthen the paper.

The use of a quasi-binomal distribution was to account for the fact the data has a high likelihood of being over or under-dispersed across the HOLC categories. We have added this rationale into the text.

3. I wonder whether the gentrification index was developed by the authors or adapted from other studies. If the authors developed it, why was "the bottom 40th percentile" chosen as a criterion? In addition, the phrase "1 of the gentrification metric (in line 237)" is confusing: so tracts eligible for gentrification satisfy at least one of the four criteria or only the first criterion?
The Gentrification index was developed by Lance Freeman in 2005, which we updated with recent data and code adapted from Vo 2008. Both these works are cited in the manuscript, but we have added more text clearly describing this as a previous developed index along with specifics mentions to each creator outside of the citation. We also see how our explanation was confusing. We clarified the text to first describe what makes a tract eligible for gentrification “median income and home value was within the bottom 40th percentile compared to tracts within the same metro area during the year 2000”; AND a tract is determined “gentrified” if it meets ALL the following criteria “median home value had increased when adjusted for inflation, the inflation-adjusted median home value placed the tract in the top third of all tracts, and the increase in college-educated adults was also in the top third of tracts within the same metro”.

Results
1. The findings described in lines 349-352 were difficult to verify through Fig. 5A. I recommend revising both the figure and the manuscript.

Thank you for the comment, on review we agree the text was not fully clear. We have revised the text for clarity and to better reflect the data displayed in Fig 5A

2. How many observations were used to estimate each statistical model in Fig. 5b (supplementary figure also)? Very few samples appear in each HOLC category, making it difficult to calculate statistical significance.

For figure 5B we are only analyzing the HOLC areas that had any tracts eligible for gentrification. This is why there are no HOLC A areas in the figure, only four HOLC B neighborhoods, and 11 HOLC C and 11 HOLC D neighborhoods. This does mean HOLC B has just enough points to calculate any statistics, though the lack of significance also reflects how little HOLC B area is eligible for gentrification. We did remove the regression lines and equations for the non-significant relationships, we also clarified in the text that this analysis is only being done on areas that are eligible for gentrification.

The supplementary figure uses all HOLC areas as points in the study (6 HOLC A, 12 HOLC B, 17 HOLC C, and 14 HOLC D

Discussion
1. More references should be used to compare or support this research's findings. Papers addressing regions beyond Denver could offer valuable implications.

We appreciate the note and we have added more recent work addressing regions outside of Denver to the introduction and to the discussion.

2. Although some limitations are mentioned at the end of the Discussion, additional methodological and conceptual limitations should be provided.

We have added a limitations section to the discussion that further describes the limitations in the work, especially highlighting that we are only able to address our research questions within the city of Denver due to the unreplicatable land-use time series we built our study from. However, we do make mention that the use of demographics as a time-series linked to HOLC categories could be used to build out similar studies in more regions.

6. PLOS authors have the option to publish the peer review history of their article ([what does this mean?](https://gcc02.safelinks.protection.outlook.com/?url=https%3A%2F%2Furldefense.com%2Fv3%2F__https%3A%2F%2Fc05y1x9s.r.us-east-2.awstrack.me%2FL0%2Fhttps%3A*2F*2Fjournals.plos.org*2Fplosone*2Fs*2Feditorial-and-peer-review-process*23loc-peer-review-history%2F1%2F010f0191ba754921-30047aee-024e-480c-a811-c18971e04b1c-000000%2FEsf9a_1h3YDNFJf5qdXRwrRc0SE%3D175__%3BJSUlJSUl!!Mak6IKo!OcFtLTvq55FlZ07cL4KFpsS8EJgEgDwCFZWTVPmz6OYupz88TWATQJqT_5CEseXq3xEA0aQsp2tIx5kp0v4%24&data=05%7C02%7Cpibsen%40usgs.gov%7Ca2a4c556dc1f4d3eabfd08dcd251e074%7C0693b5ba4b184d7b9341f32f400a5494%7C0%7C0%7C638616496374322990%7CUnknown%7CTWFpbGZsb3d8eyJWIjoiMC4wLjAwMDAiLCJQIjoiV2luMzIiLCJBTiI6Ik1haWwiLCJXVCI6Mn0%3D%7C0%7C%7C%7C&sdata=IX%2FwqvoBv2rdyeFc4g%2FI64eMNnJ7rYSMCijEFRgsJHU%3D&reserved=0)). If published, this will include your full peer review and any attached files.


If you choose “no”, your identity will remain anonymous but your review may still be made public.


**Do you want your identity to be public for this peer review?** For information about this choice, including consent withdrawal, please see our [Privacy Policy](https://gcc02.safelinks.protection.outlook.com/?url=https%3A%2F%2Furldefense.com%2Fv3%2F__https%3A%2F%2Fc05y1x9s.r.us-east-2.awstrack.me%2FL0%2Fhttps%3A*2F*2Fwww.plos.org*2Fprivacy-policy%2F1%2F010f0191ba754921-30047aee-024e-480c-a811-c18971e04b1c-000000%2FFqajDsAAEpdxIvdoinCuOIGvIWw%3D175__%3BJSUl!!Mak6IKo!OcFtLTvq55FlZ07cL4KFpsS8EJgEgDwCFZWTVPmz6OYupz88TWATQJqT_5CEseXq3xEA0aQsp2tI2RgbS1Q%24&data=05%7C02%7Cpibsen%40usgs.gov%7Ca2a4c556dc1f4d3eabfd08dcd251e074%7C0693b5ba4b184d7b9341f32f400a5494%7C0%7C0%7C638616496374330078%7CUnknown%7CTWFpbGZsb3d8eyJWIjoiMC4wLjAwMDAiLCJQIjoiV2luMzIiLCJBTiI6Ik1haWwiLCJXVCI6Mn0%3D%7C0%7C%7C%7C&sdata=gdXUKue9uOKYN%2BnFfvniMZ0ov7w0qtWPr%2BdcqYf87DQ%3D&reserved=0).

Reviewer #1: No

Reviewer #2: No
